# Patterns of noise exposure and prevalence of hearing loss amongst Cape Town Minstrel Carnival musicians

**DOI:** 10.4102/sajcd.v68i1.789

**Published:** 2021-05-31

**Authors:** Lebogang Ramma

**Affiliations:** 1 Department of Health and Rehabilitation Sciences, Faculty of Health Sciences, University of Cape Town, Cape Town, South Africa

**Keywords:** noise-induced hearing loss (NIHL), musicians, minstrel carnival, noise exposure, prevalence

## Abstract

**Background:**

Cape Town Minstrel Carnival is one of the oldest and most authentic indigenous New Year’s customs in South Africa. Musicians who perform at this carnival are exposed to excessively loud music and therefore at a risk of acquiring noise-induced hearing loss (NIHL).

**Objectives:**

This study aimed to determine patterns of exposure to loud music and prevalence of hearing loss amongst Cape Town Minstrel Carnival musicians.

**Method:**

A descriptive, observational exploratory survey design was used and 43 participants (21 males and 22 females; mean age, 21 ± 9 years) took part in this study. Sound level measurements were conducted to assess musicians’ sound exposure during rehearsals and performances. All participants underwent the following audiological test battery at least 2 h before music exposure: Case history, otoscopic examination, tympanometry, pure tone audiometry and distortion products otoacoustic emission (DPOAE).

**Results:**

Average noise levels recorded were 86 dBA during rehearsals and 98.7 dBA at performances and average durations of exposure were 240 and 10 min at rehearsals and performances, respectively. One out of 43 (1/43) participants presented with sensorineural hearing loss. Audiometric results of the remaining participants were normal and did not show a pattern suggestive of NIHL. A high proportion of participants (21/43) reported experiencing tinnitus.

**Conclusion:**

Despite being exposed to high levels of noise, there was a low prevalence of hearing loss amongst these musicians. However, a high proportion of them reported tinnitus, which could be an indication that they were at a high risk of NIHL from the music that they played.

## Introduction

The Cape Town Minstrel Carnival is one of the oldest and most authentic indigenous New Year’s customs in South Africa (Martin, 2007). At the beginning of every year, scores of minstrel troupes, locally known as *Kaapse Klopse* [Clubs of the Cape] partake in the carnival, which include Christmas bands and Malay choirs (Oliphant, 2013). The first and second of January of every year usually represent the apex of the carnival, with performances from early in the morning until the early hours of the next days. However, performances continue every weekend throughout the month of January and beyond (Bruinders, 2007).

Troupes typically consist of a few hundred members, mostly non-professional musicians who perform at the carnival as a pastime, with some members assigned as captains and into respective committee roles (Martin, 1999). People of various age groups form part of the troupes, ranging from young toddlers to elderly over 70 years, with the bulk of the troupe members consisting of teenagers and young adults. Members perform various roles, including singing, dancing and playing instruments. The music played by the troupes typically includes light Western, pop and jazz music; folk songs; and their own compositions (Martin, 1999). Instruments often include string instruments, like guitars and banjos, drums, tambourines and brass instruments (Martin, 1999).

Each troupe has a brass band that consists of 20–30 musicians, with more vocalists and dancers than musicians. Members of the troupes usually practice up to three times per week for several months per year and rehearsal venues vary from garages, back yards, community halls and houses across the suburbs of Cape Town (Baxter, 2001). During brass band rehearsals, music exposure is non-continuous and many silent breaks are granted. At competitions, the duration of performances typically range from 10 to 20 min per performance.

There is a growing body of evidence that suggests that exposure to loud music and recreational noise for extended periods of time poses a risk of temporary or permanent noise-induced hearing loss (NIHL) (Le Clercq, Van Ingen, Ruytjens, & Van der Schroeff, 2016; Le Prell, et al. 2012; Neitzel & Fligor, 2019) and tinnitus (Guest, Munro, Prendergast, Howe, & Plack, 2017; Zhao, Manchaiah, French, & Price, 2010) to those exposed. Events such as the Cape Town Minstrel Festival generate high levels of noise and therefore pose the risk of NIHL to those who participate, especially performers. Noise-induced hearing loss can have a negative impact on many aspects of an individual’s life, social and educational development and their ability to work (World Health Organization, 2015), and therefore should be prevented at all costs.

Noise-induced hearing loss resulting from music exposure, especially amongst professional musicians, has been documented in several studies (Halevi-Katz, Yaakobi, & Putter-Katz, 2015; Pawlaczyk-Łuszczyńska, Dudarewicz, Zamojska, & Śliwinska-Kowalska, 2011; Pouryaghoub, Mehrdad, & Pourhosein, 2017; Schink et al., 2014). Some of these studies have reported a high prevalence of NIHL amongst professional musicians (Emmerich, Rudel, & Richter, 2008; Phillips, Henrich, & Mace, 2010; Pouryaghoub et al., 2017; Schink et al., 2014). Relatively fewer studies have investigated the prevalence of NIHL amongst non-professional musicians. Few studies that investigated NIHL amongst non-professional musicians have reported almost no NIHL amongst these musicians although they were also exposed to noise levels that could lead to NIHL (Schmuziger, Patscheke, & Probst, 2006; Virmond, Perroca, & Moura, 2007).

There is some evidence that the content of the sound and the listener’s subjective perception of sound exposure may affect the risk of a temporary threshold shift (TTS) (Strasser, Irle, & Scholz, 1999). This was further shown in a study by Strasser and colleagues in which it was demonstrated that the amount of TTS varied according to the type of sound one is exposed to (Strasser, Irle, & Legler, 2003). For instance, it was shown in the same study that exposure to loud classical music led to substantially less TTS than industrial noise of the same duration and intensity (Strasser et al., 2003). This has subsequently led to some researchers (Virmond et al., 2007) questioning whether exposure to music can cause NIHL amongst non-professional musicians.

Virmond and colleagues posited that there is a possible existing association between the perception of music as a pleasant sound and some psychophysiological protective effect (Virmond et al., 2007). That is, a positive attitude from musicians towards their music may explain why non-desirable sound of similar intensity levels and duration may lead to a more damaging effect on hearing than their own music does (Virmond et al., 2007). In addition, music and industrial noise are different in terms of their spectral composition and this may also explain the differences in their overall risk to human hearing (Kähäri, Axelsson, Hellstrom, & Zachau, 2001; Virmond et al., 2007). However, Ivory, Kane and Diaz (2014) reported that music, even when used recreationally, can cause damage to the auditory system, which seems to contradict views of Virmond et al. (2007) on this issue.

Whilst the duration and intensity of the sound are usually used as key indicators to judge the risk of NIHL, it is important to bear in mind that these variables (intensity and duration) interact with other factors in influencing an individual’s susceptibility to hearing loss. Amongst others, factors such as age, gender, previous non-music-related noise exposure, type of instruments played and accumulated music exposure can also increase a person’s susceptibility to developing hearing loss (Agrawal, Platz, & Niparko, 2009; Daniel, 2007).

Although there are occupational regulations and standards developed to protect individuals who work in excessively noisy environments from acquiring NIHL, musicians are often overlooked when it comes to occupational safety and health practices (National Institute for Occupational Safety and Health [NIOSH], 2015). This is especially true for non-professional musicians who may not even consider themselves to be at risk of NIHL. For instance, Pouryaghoub et al. (2017) reported that most musicians do not have adequate knowledge about hearing conservation and therefore, most of them never use protective devices to prevent NIHL. Also, despite musicians being at risk of many auditory disorders because of exposure to loud music, most of them are less concerned about these issues and therefore do not use hearing protection (Dinakaran, Deborah, & Thadathil, 2018; Santucci, 2010).

Musicians in the Minstrel troupes, especially those who play brass instruments, are exposed to high-intensity music levels for extended periods during rehearsals and performances. Therefore, these musicians are at risk of developing NIHL from prolonged exposure to high levels of noise. The aim of this study was to determine the patterns of noise exposure and the prevalence of hearing loss amongst Cape Town Minstrel Carnival musicians.

## Methods

### Study design, participants and setting

A descriptive, observational exploratory survey design was used in this study. Participants were members of the Minstrel troupes in the Cape Town metropolitan area and were sampled via convenience sampling. To be included in this study, participants had to be between 5 and 49 years old to minimise the impact of age-related hearing loss (De Sousa, Júnior, Larsson, & Ching, 2009). Participants who reported history of work-related noise exposure, prior treatment with ototoxic medications, middle ear pathologies and history of ear surgery were excluded from the study.

### Data collection instruments and procedures

The study proposal was first submitted to the University of Cape Town, Faculty of Health Sciences Human Research Ethics Committee (HREC) for ethics review and approval. After obtaining ethics approval, Minstrel troupes were contacted and invited to take part in the study. Appointments for data collection were arranged with the groups that agreed to participate. On the arranged date of data collection, the researchers met members of the troupe, explained the purpose of the study and what participation would entail. Participants were asked to indicate their willingness to take part in this study by signing an informed consent form (assent was obtained from participants younger than 18 years old). Audiometric assessment took place in carefully selected quiet rooms (ambient noise levels < 50 dB SPL), using noise-attenuating headphones at each of the rehearsal venues. Audiometric assessment was conducted at least 2 h prior to the start of rehearsal sessions.

Audiological assessment included the following: Case history interview (with emphasis on prior history of noise exposure), otoscopy, tympanometry (GSI 39) (Gradson-Stadler, United States of America), pure tone audiometry (2 kHz – 8 kHz) (KuduWave 5000 Audiometer) (GeoAxon, South Africa) and distortion products otoacoustic emissions (DPOAEs) (GSI Audera V2.7) (Gradson-Stadler, United States of America). Parameters for DPOAE are set as follows: L1 = 65 dB and L2 = 55 dB, with an intensity ratio of L1–L2 = 10 dB, and frequency range: 2098, 2402, 2754, 3152, 3621, 4184, 4816, 5496, 6340 and 7277 Hz. For a DPOAE response to be considered normal at a given frequency, it would have to be 3 dB or more above noise floor and absolute distortion product (DP) amplitude had to be greater than -10 dB (Hall & Swanepoel, 2010). Participants who showed abnormal audiometric findings were counselled about their test results and were referred to the nearest public healthcare facility for further management.

Sound level measurements were carried out using a Brüel & Kjær (B&K) (Bremen, Germany) Type 2236 sound level meter (SLM). The SLM was calibrated before and after each measurement using the manufacturer’s provided calibrator and then compared against a sound source with known intensity level. Sound level meter was also fitted with a manufacturer supplied windscreen to minimise impact of wind noise, which could introduce errors in the recorded sound levels. Consideration for the impact of wind in sound measurements was especially important when conducting measurements during performances because those were performed outdoors. The SLM is set as follows: a-scale frequency weighting, a slow time weighting and a range of 70 dB – 140 dB.

Measurements were conducted during the troupe’s rehearsals and at performances during many of the minstrel activities, including the final competition at the Athlone Stadium in Cape Town. During rehearsals, sound levels were measured from different points across the room to obtain sound level samples from musicians sitting in the front, middle and back of the room. The SLM was placed on a tripod stand, set at least 1.2 m above the floor and at least 1.5 m away from large reflecting surfaces (e.g. the walls). The measurements were recorded at 5 min intervals (i.e. at least 5 min for each interval of recording). During performances, measurements were recorded from between the two rows of musicians as they marched, also ensuring to obtain noise samples from the back, middle and front of the row. The SLM was held at an arms length from the individual doing the recording, at about ear level (whilst standing) of the musicians. Several samples of sound level measurements were recorded and only peak and equivalent continuous average (L_Aeq_) sound levels were recorded and reported for each sound measurement session.

### Data analysis

Data were analysed using both descriptive statistics (mean, standard deviations, range) and inferential statistics. For inferential statistics, the Mann-Whitney U test was used to assess whether there was a statistically significant difference (*p* = 0.05) between the means of different categories of participants. Data were analysed using Stata 11.1 statistical software package (StataCorp, United States of America).

### Ethical considerations

Ethical approval for this study was granted by the University of Cape Town, Faculty of Health Sciences HREC (No. 556/2011). Ethical conduct during this study was in accordance with and guided by the Declaration of Helsinki as amended by the 64th World Medical Association, Fortaleza, Brazil, in 2013.

## Results

A total of 43 participants took part in this study. Participants were divided into two categories in accordance with their primary roles in the troupe: instrument players (instrumentalists) or singers (vocalists). There were more instrumentalists than vocalists; however, vocalists were generally older and had more musical experience (ME) than instrument players. All participants reported that they had never used hearing protection devices to prevent potential NIHL from the music they played (Table 1).

**TABLE 1:** Participants’ description (*n* = 43).

Variable	Instrumentalists (*n* = 31)	Vocalists (*n* = 12)
Mean age (years)	16 ± 1	30 ± 3
**Sex**		
Males	16	5
Females	15	7
History of tinnitus	14	5
Mean band membership duration (years)	5 ± 2	7 ± 3
Mean weekly music exposure (hours)	4 ± 2	7 ± 4
Mean musical experience (ME)	23 ± 17	52 ± 41
Participants playing trumpet	15	-
Participants playing other instruments	16	-

Note: Musical experience = years of playing the instruments × hours of weekly music exposure.

These musicians were exposed to loud musical sounds for longer durations during rehearsals and to excessively loud sound levels (but for shorter durations) during musical performances at many of the Minstrel festivities (Table 2).

**TABLE 2:** Sound levels that (dBA) musicians were exposed to during rehearsals and performances.

Recorded noise levels	L_Aeq_ (dBA)	Median range	Peak sound level (dBA)	Average duration of exposure (min)	Safe exposure duration (min)†
Rehearsals	86	83.5–88	111	240	240–960
Performances	98.7	95–102	124.2	10	7.5–30

† , Safe exposure duration was determined using NIOSH (2015). Recommended exposure level (REL) of 85 dBA for 8 h at an exchange rate of 3 dB.

Two participants in the vocalist group (2/12) displayed abnormal results for both pure tone audiometry and DPOAE assessment. However, one of these two participants also had an abnormal (Type B) tympanogram, which was consistent with a middle ear pathology. The hearing test results of the participant with Type B tympanogram were not included in further analysis of results. All the participants in the instrumentalist group passed pure tone screening and DPOAE assessment. Furthermore, the average hearing thresholds (4 kHz – 8 kHz) of the participants were all within normal limits and did not show any pattern consistent with exposure to excessive noise (see Table 3).

**TABLE 3:** Mean hearing thresholds of instrument players only (*n* = 31) at 4.6 kHz and 8 kHz.

Hz	Right ear		Left ear	
	Mean	Standard deviation	Mean	Standard deviation
4000	17.58	7.19	16.21	6.61
6000	15.45	8.78	13.64	9.03
8000	13.18	9.08	13.03	10.6

**FIGURE 1:**
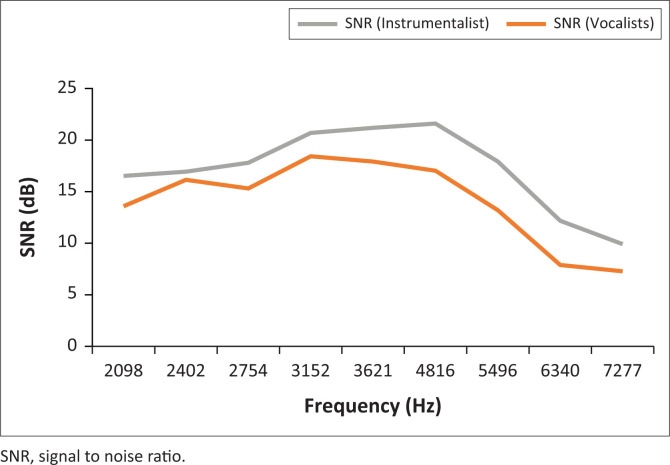
Distortion products otoacoustic emission signal to noise ratio for instrumentalists and vocalists (left ear).

Distortion products otoacoustic emission results showed that responses of participants in the instrumentalist group had higher signal-to-noise ratio (SNR) when compared with those in the vocalist group. However, the response of both groups showed a similar trend (more robust in the mid frequencies and weaker in the higher frequencies) (see Figure 1).

Mann–Whitney U test (*p* < 0.05) revealed no statistically significant difference in DPOAE amplitudes between the following: instrumentalists versus vocalists (*p* = 0.90), trumpet players versus other instrument players (*p* = 0.84), weekly noise exposure (< 4 h/week vs. > 4 h/week) (*p* = 0.63) and ME (< 20 vs. > 20) (*p* = 0.84). Finally, there was also no statistically significant difference between the average DPOAE amplitudes of vocalists and that of instrumentalists at any of the tested frequencies.

## Discussion

This study set out to investigate the patterns of exposure to loud music by musicians who perform in the annual Cape Town Minstrel Carnival and to determine the prevalence of hearing loss amongst these musicians. Overall findings of the study showed that Cape Town Minstrel Carnival performers are exposed to excessively loud sounds during both rehearsal sessions (> 85 dBA) and performances (> 98 dBA). A high proportion of them reported experiencing tinnitus; however, there was a very low prevalence of hearing loss amongst these musicians.

In terms of the patterns of noise exposure, it was observed that these musicians are exposed to loud music for a much longer duration during rehearsal sessions (on average, 4 h per session three times per week, during intensive preparation period) than during performances. Exposure to high levels of sound during performances was surprisingly very brief (on average, 10 min per session). However, musicians will typically warm up (10–15 min) before going on stage for performances. Noise levels that these musicians were exposed to at both rehearsals and performances exceeded the NIOSH’s recommended exposure level (REL) for musicians (NIOSH, 2015), which means that they are at risk of developing NIHL. Patterns of noise exposure and noise levels that musicians in this study were routinely exposed to were consistent with those reported in previous studies involving musicians who play similar types of instruments (i.e. brass instruments) (Emmerich et al., 2008; Schmidt et al., 2011). Similar noise exposure patterns were also reported by Mcllvaine and colleagues, that is, noise levels exceeding NIOSH recommended noise levels, with higher noise intensities recorded during performances than rehearsals (McIlvaine, Stewart, & Anderson, 2012).

Given the high noise levels that these musicians were routinely exposed to, it was surprising to find that only one of the 43 participants in this study presented with a hearing loss (mild high frequency sensory-neural hearing loss). For the remainder of the participants, average pure tone hearing thresholds test results were not consistent with what would be expected in a population that is regularly exposed to these noise levels. Some researchers have cautioned about the risk of pure tone audiometry underestimating noise-induced hidden hearing loss because it is often not accompanied by a shift in pure tone hearing thresholds (Shi et al., 2016). It was for this reason that DPOAE was included as part of the test battery. However, average DPOAE amplitudes were also found to be within the normal range at all the frequencies tested, regardless of the role or the musical instrument that the musicians played in the troupe.

The fact that there were fewer musicians in this study who presented with hearing loss was an unexpected finding, especially when considering that all the participants reported that they had never used hearing protection devices during rehearsals or performances. However, this was consistent with the findings of previous studies that investigated prevalence of hearing loss amongst non-professional pop musicians, which also reported no hearing loss amongst this group of musicians (Schmuziger et al., 2006; Virmond et al., 2007). One of the explanations given for the absence of hearing loss amongst these musicians was because musicians generally perceive music as pleasant and this is thought to have a psychological protective effect that reduces the risk of NIHL amongst them (Virmond et al., 2007).

Specific to this study, it was observed that whilst these musicians were exposed to loud music for longer durations during rehearsals, there were often many breaks in the music that they were exposed to. For instance, during the practice of a new song, there were frequent breaks in the music to allow other players to master the song to be performed. These frequent pauses are said to be protective when compared with a continuous noise, such as industrial noise, which tends to lead to more hearing loss (Virmond et al., 2007). Furthermore, Cape Town Minstrel musicians do not play any amplified music. Amplified music has been shown to present a much higher risk of NIHL than unamplified music (Halevi-Katz et al., 2015). It was also observed that musicians who participated in this study, especially those who played instruments, were generally young (average age of 16 years) and most of them have been involved in this kind of activity for a short period of time (< 5 years). This could potentially explain why there was such a low prevalence of hearing loss in this group.

It was noticed that a high proportion of participants in this study reported experiencing tinnitus: 14/31 (instrumentalists) and 5/12 (vocalists). Tinnitus is known to be closely associated with music exposure and indicates a high risk of an individual developing NIHL as a result of music exposure (Zhao et al., 2010). However, because of the limited number of questions related to tinnitus, it was not possible to further probe participants who reported tinnitus to ascertain whether they were reporting transient tinnitus or tinnitus that interfered with their daily functioning.

The fact that most participants in this study reported not using hearing protection when exposed to loud music was expected, especially when considering the informal nature of their musical activities. Musicians generally lack awareness about hearing conservation to prevent NIHL (Pouryaghob et al., 2017; Dinakaran et al., 2018) and therefore do not usually follow proper hearing conservation practices (Santucci, 2010).

The findings of this study should be interpreted whilst taking into account its methodological limitations; this study did not assess noise dose of individual musicians during both rehearsals and performances, which could have given a more detailed NIHL risk analysis of individual musicians. Instead, noise levels and exposure patterns were assessed over a period of 6 months to obtain a better understanding of how these musicians interact with noise. Furthermore, this study involved a small sample size and participants were not randomised when enrolled to the study, which limited generalisability of this study. However, despite its methodological shortcomings, this study is one of the first studies to investigate the prevalence of hearing loss and the risk of NIHL in this group of musicians. This study has objectively shown that these musicians are indeed at risk of developing NIHL as a result of their music exposure.

## Conclusion

Cape Town Minstrel musicians are often exposed to sound levels that are above the NIOSH REL during both rehearsals and performances. Despite this and not using any form of hearing protection, this study found a very low prevalence of hearing loss in this group of musicians. Both audiometric and DPOAE test results did not indicate any pattern consistent with NIHL. However, a high proportion of these musicians reported tinnitus, which may be an indication of increased risk of NIHL amongst them. It is therefore very important that these musicians, who are central to this carnival, are made aware of the potential risk the music they play impose to their hearing status and are educated about viable and sustainable strategies such as the use of personal hearing protection devices to prevent potential NIHL from their musical activities.
